# Resistance of advanced cassava breeding clones to infection by major viruses in Uganda

**DOI:** 10.1016/j.cropro.2018.09.015

**Published:** 2019-01

**Authors:** Daniel Rogers Mukiibi, Titus Alicai, Robert Kawuki, Geoffrey Okao-Okuja, Fred Tairo, Peter Sseruwagi, Joseph Ndunguru, Elijah Miinda Ateka

**Affiliations:** a Jomo Kenyatta University of Agriculture and Technology, P.O. Box 62000-00200, Nairobi, Kenya; b National Crops Resources Research Institute, P.O. Box 7084, Kampala, Uganda; c Mikocheni Agricultural Research Institute, P.O. Box 6226, Dar es Salaam, Tanzania

**Keywords:** Cassava brown streak disease, Cassava mosaic disease, Tolerance, Incidence, Severity, Virus load

## Abstract

Cassava brown streak disease (CBSD) and cassava mosaic disease (CMD) are two viral diseases that cause severe yield losses in cassava of up to 100%, thereby persistently threatening food and income security in sub-Saharan Africa. For effective management of these diseases, there is a critical need to develop and deploy varieties with dual resistance to CBSD and CMD. In this study, we determined the response of advanced breeding lines to field infection by cassava brown streak viruses (CBSVs) and cassava mosaic begomoviruses (CMBs). This aim helped in identifying superior clones for downstream breeding. In total, 220 cassava clones, three in uniform yield trials (UYTs) and 217 in a crossing block trial (CBT), were evaluated for virus and disease resistance. Field data were collected on disease incidence and severity. To detect and quantify CBSVs, 448 and 128 leaf samples from CBSD symptomatic and symptomless plants were analyzed by reverse transcription PCR and real-time quantitative PCR, respectively. In addition, 93 leaf samples from CMD symptomatic plants in the CBT were analyzed by conventional PCR using CMB species-specific primers. In the CBT, 124 (57%) cassava clones did not express CMD symptoms. Of the affected plants, 44 (55%) had single *African cassava mosaic virus* infection. Single *Cassava brown streak virus* (CBSV) infections were more prevalent (81.6%) in CBT clones than single *Ugandan cassava brown streak virus* (UCBSV) infection (3.2%). Of the three advanced clones in the UYT, NAROCASS 1 and NAROCASS 2 had significantly lower (*P* < 0.05) CBSD severity, incidence, and CBSV load than MH04/0300. In the UYT, only 22% of samples tested had CBSVs, and all showed a negative result for CMBs. The low disease incidence, severity, and viral load associated with NAROCASS 1 and NAROCASS 2 is evidence of their tolerance to both CBSD and CMD. Therefore, these two cassava clones should be utilized in CBSD and CMD management in Uganda, including their utilization as progenitors in further virus resistance breeding.

## Highlights

• Host plant resistance is essential for management of cassava mosaic disease (CMD) and cassava brown streak disease (CBSD).
• Disease evaluation based on symptoms need to be augmented with molecular tools for better disease assessment.
• *Cassava brown streak virus* is the most prevalent virus causing CBSD in Uganda.

## 1 Introduction

Cassava brown streak disease (CBSD) and cassava mosaic disease (CMD) have persisted as major constraints to the production of cassava (*Manihot esculenta* Crantz) in sub-Saharan Africa (SSA), where the crop serves as a major staple food (Alicai et al., 2007; Mohammed, 2012). Storage roots of plants affected by CBSD have a brown necrotic rot and are unfit for consumption. Furthermore, the starch content of CBSD-affected storage roots is greatly reduced and of inferior quality (Nuwamanya et al., 2015). By contrast, storage roots of plants severely affected by CMD fail to bulk because their leaves become chlorotic and mottled, thus having impeded photosynthesis and leading to stunted growth (Mohammed, 2012). Both viral diseases are perpetuated from one season to another through the practice of farmers using stem cuttings from diseased plants as planting material (Hillocks and Jennings, 2003). Farmers in Uganda and Africa generally obtain cassava planting materials from stems of their previous crop or neighbors’ fields. The virus status of the planting materials is often unknown because symptoms may not be apparent or leaves may have withered and dropped after crop harvest. This practice leads to the spread of viral diseases to new fields and often advances into disease epidemics. This commonly results in yield losses of up to 100% due to CBSD and CMD, either singly or in combination, thus threatening food security in the region (Hillocks, 2003). In addition, the year-round occurrence of high whitefly (*Bemisia tabaci*) vector populations in many parts of eastern Africa escalates the spread of CMD and CBSD.

CBSD is caused by two single-stranded RNA viruses of the genus *Ipomovirus*, *Cassava brown streak virus* (CBSV) and *Ugandan cassava brown streak virus* (UCBSV), often jointly referred to as CBSVs. Both viruses belong to the family Potyviridae (Mbanzibwa et al., 2009; Winter et al., 2010). The most prevalent viruses that are causal agents of CMD in SSA are single-stranded DNA bipartite cassava mosaic begomoviruses (CMBs), *African cassava mosaic virus* (ACMV) and *East African cassava mosaic virus* (EACMV), of the family Geminiviridae (Bock and Woods, 1983; Swanson and Harrison, 1994). All these viruses are transmitted by *B. tabaci* (Maruthi et al., 2005), and the disease is spread through the use of infected planting materials. The high demand for cassava planting materials and the lack of functional seed systems for the crop have resulted in the movement of uncertified stem cuttings to be used as planting materials across communities, thereby increasing the spread of the viral diseases. Unless CBSD is controlled through appropriate management strategies, it could potentially become a menace across SSA, including West Africa where the major cassava-producing countries are located.

Effective management of CBSD and CMD will heavily rely on the deployment of resistant varieties to farmers. Fortunately, concerted efforts on the genetic improvement of cassava in Uganda have generated large breeding populations with several elite clones deemed as either tolerant or resistant to both CBSD and CMD (Kawuki et al., 2016). Importantly, genotypes with durable dual resistance to CBSD and CMD should be a target for breeders when screening clones for variety selection. Thus, it is imperative that breeding clones are selected for advancement considering resistance to virus signified by the viral load in plant tissues and the response of genotypes to the diseases under field conditions. Therefore, in this study, we determined resistance or tolerance to infection by CBSVs and CMBs (and resulting CBSD and CMD) in a panel of elite breeding clones, and this is critical for facilitating the development of resistant cassava varieties.

## 2 Materials and methods

### 2.1 Test materials and study design

The test materials evaluated in this study include elite clones under evaluation in uniform yield trials (UYTs) and the breeding lines in the crossing block trial (CBT) conducted from 2014 to 2015. The CBT was performed at Namulonge, Wakiso district (Central Uganda), and UYTs were performed at the four major cassava-producing districts: Arua (north-western), Kamuli (eastern), Kaberamaido (north-eastern), and Wakiso. Each of the test sites was located in unique agro ecology, thus differing in cassava viral disease pressure, vector population, and major climatic parameters including mean annual temperature and rainfall. Kaberamaido is located at an elevation of 1080 m.a.s.l., with an annual average temperature of 23.7 °C and annual average precipitation of 1302 mm. Kamuli has an elevation of 1100 m.a.s.l., with an annual average temperature of 21.6 °C and annual average precipitation of 968 mm. Arua is at an elevation of 1215 m.a.s.l. and is characterized by an annual average temperature of 22.9 °C and annual precipitation of 1404 mm. Wakiso has an elevation of 1200 m.a.s.l., with an annual average temperature of 21.8 °C and annual average precipitation of 1377 mm.

Three clones (NAROCASS 1, NAROCASS 2, and MH04/0300) were evaluated in the UYTs for resistance to CBSD and CMD and adaptation to the locations. The officially released CBSD-tolerant variety NASE 14 was included as a control. The NAROCASS 1 clone bred as NAM 130 in Uganda was a selection made from open-pollinated seeds introduced from Tanzania. The NAROCASS 2 clone whose pedigree is MM2006/0130 was bred as MM06/0130 in Uganda. NASE 14, a genotype of pedigree MM96/4271 and bred as MM192/0248, is an IITA introduction officially released in Uganda. Experimental plot sizes were 6 m × 6 m, laid out in a randomized complete block design with four replications per site and plant spacing of 1 m × 1 m. In the CBT, a total of 217 cassava breeding lines, which were part of NaCRRI cassava training population being used in genomic selection, were planted as single-row plots of 10 plants and monitored for disease severity and incidence. To increase the amount of CBSV inoculum, infector rows of the CBSD-susceptible variety TME 204 were planted between test plots. Disease incidence and severity data were collected at 3, 6, and 12 months after planting (MAP).

### 2.2 Field assessment of CBSD and CMD

The CBSD and CMD severities were assessed and recorded for each cassava plant in the trials at 5, 8, and 11 MAP. CBSD symptom severity was scored on a scale with a rating from 1 to 5 points: 1 = no apparent symptoms; 2 = slight foliar feathery chlorosis and no stem lesions; 3 = pronounced foliar feathery chlorosis, mild stem lesions, and no dieback; 4 = severe foliar feathery chlorosis, severe stem lesions, and no dieback; and 5 = defoliation, severe stem lesions, and dieback (Gondwe et al., 2003). The CMD was scored on a scale of with a rating from 1 to 5 points: 1 = no symptoms observed (shoot healthy); 2 = mild chlorotic pattern on most leaves, mild distortions at the bases of most leaves, with the remaining parts of the leaves and leaflets appearing green and healthy; 3 = a pronounced mosaic pattern on most leaves, with narrowing and distortion of the lower one-third of most leaves; 4 = severe mosaic distortion of two-thirds of most leaves, with general reduction in leaf size and some stunting of shoots; and 5 = very severe mosaic symptoms on all leaves, with distortion, twisting, and severe reduction in leaf size in most leaves, accompanied by severe stunting of plants (IITA, 1990). The CBSD and CMD incidence data were obtained from the number of plants showing foliar disease symptoms, expressed as a percentage of the total number of plants assessed.

### 2.3 Determination of CBSD root severity, harvest index, and yield in the UYT

The CBSD root severity was assessed by making five cross-sectional cuts with a knife on all the roots and scoring necrosis for each cut section using a pictorial severity scale (rated with 1–5 points) as described by Hillocks and Thresh (2000). The harvested roots together with the ground biomass above were weighed separately, and the weights were used to compute the harvest index (HI) as the ratio of weight of the roots to the total biomass. Yield was estimated using the formula.

Root yield (t/ha) = [root weight (kg/m^2^) × 10000]/1000 (Kamau et al., 2011).

Analysis of variance was performed with the mean values of CBSD root incidence, severity, HI, and yield of cassava clones in the UYT to assess whether significant differences existed among the test clones.

### 2.4 Sample collection for laboratory detection of CBSVs and CMBs

For detection of CBSVs, 448 leaf samples (88 symptomatic and 360 non-symptomatic) were collected from the UYT plots. Non-senescing leaves in the middle to bottom canopy section on a shoot representative of the plant stand were collected. Additionally, a total of 93 CMD-symptomatic leaf samples were collected from the CBT for detection of CMBs. Each sample was placed in a 1.5-ml microfuge tube containing 70% ethanol and stored at room temperature until DNA extraction.

### 2.5 RNA extraction and RT-PCR for CBSV detection

RNA was extracted from each leaf sample by a modified cetyl trimethyl ammonium bromide (CTAB) method described by Chang et al. (1993). The resultant RNA pellets were dried at room temperature and then resuspended in 50 μl of autoclaved nuclease-free water and stored at −80 °C until analysis. cDNA was synthesized from each of the RNA extracts using SuperScript™ III Reverse Transcriptase kit (Invitrogen, Carlsbad, CA, USA). The generated cDNAs were subjected to RT-PCR for simultaneous detection of CBSV and UCBSV.

The PCR master mix was prepared in a PCR reaction tube, and 2 μl of the cDNA template was added to this master mix to constitute a final reaction volume of 50 μl. The components of the PCR master mix are as follows: 38.1 μl of sterile distilled water; 5 μl of 10 × PCR buffer [200 mM Tris-HCl (pH 8.4) and 500 mM KCl]; 1.5 μl of 50 mM MgCl_2_; 1 μl of 10 mM dNTP mix; 1 μl of amplification forward primer CBSDDF2 (5′-GCTMGAAATGCYGGRTAYACAA-3′) (10 μM) and 1 μl of amplification reverse primer CBSDDR (5′-GGATATGGAGAAAGRKCTCC-3′) (10 μM), which were degenerate primers for CBSV and UCBSV (Mbanzibwa et al., 2011); and 0.4 μl of Taq DNA polymerase (5 U/μl). The following PCR thermocycling conditions were used: 94 °C for 5 min for initial denaturation; 94 °C for 30 s and 51 °C for 30 s for one cycle; and then 35 cycles of 94 °C for 30 s, 51 °C for 30 s, and 72 °C for 30 s for denaturation, annealing, and extension, respectively. The final extension was at 72 °C for 10 min. The PCR products for the detection of CBSVs were separated by electrophoresis in 1.0% agarose gels stained with ethidium bromide at 80 V for 40 min in 1 × Tris-Acetate-EDTA (TAE) buffer at pH 8.0. Gels were visualized under UV light and photographed using a digital camera.

### 2.6 DNA extraction and PCR for the detection of CMBs

To enable the detection of CMBs, DNA was extracted from leaf samples using the method described by Dellaporta et al. (1983). The DNA was initially purified by solvent extraction using a solution of phenol:chloroform:isoamyl alcohol (25:24:1) and finally by a mixture of ethanol and sodium acetate solution. Resultant pellets were washed with 70% ethanol, dried, and resuspended in 200 μl of deionized and autoclaved distilled water and stored at −80 °C until use.

To detect CMBs, DNA extracts were amplified by standard PCR using ACMV and EACMV-UG2 specific primers described by Harrison et al. (1997): ACMV-AL1/F and ACMV-ARO/R as well as ACMV-CP/R3 and UV-AL1/F1, respectively. PCR products from the DNA of CMBs were separated by electrophoresis in a 1.2% agarose gel stained with ethidium bromide at 85 V for 1 h in TAE buffer at pH 8. The gels were visualized under UV light and photographed using a digital camera. Band sizes were used to identify each virus species.

### 2.7 Determination of CBSV and UCBSV titers with real-time quantitative PCR (qPCR)

To determine viral load of CBSVs using qPCR, total RNA was extracted from leaf samples by the CTAB method following the manufacturer's protocol as described by Lodhi et al. (1994) with modifications by Ogwok et al. (2012). The extracted RNA was suspended in 50 μl of nuclease-free water and stored at −20 °C. The RNA was cleaned of cassava genomic DNA using DNAse I digestion according to the manufacturer's recommendation (Life Technologies, Grand Island, NY, USA). Quantity, purity, and integrity of each RNA sample were assessed using NanoDrop 2000c spectrophotometer (Thermo Fisher Scientific, Waltham, MA, USA) and visually by electrophoresis on a 1.5% agarose gel. Only samples with OD readings (260/280 nm) of 1.8–2.2 were used for qPCR. Two micrograms of each RNA sample was converted into cDNA using a ProtoScript^®^ First-Strand cDNA synthesis kit primed with oligo-dT following the manufacturer's protocol. qPCR was performed using SYBR Green I chemistry on an Applied Biosystems StepOnePlus^®^ sequence detection system using 96-well reaction plates with two or three technical replicates of each sample. The total reaction volume of 10 μl was used in each well with 5 μl of Thermo Scientific Maxima SYBR Green/ROX qPCR Master Mix (2 × ), 1 μl of each primer pair CBSVqF/CBSVqR (Fwd 5′-GCCAACTARAACTCGAAGTCCATT-3′ and Rev 5′-TTCAGTTGTTTAAGCAGTTCGTTCA-3′) (Adams et al., 2013) and UCBSVqF/UCBSVqR (Fwd 5′-AAGGCAAGGGTGGCTCTAAC-3′ and Rev 5′-GCGTCCTTGTTGGCCCATTA-3′), and 3 μl of template cDNA. Cytochrome C oxidase COX F/COX R (Fwd 5′-CGTCGCATTCCAGATTATCCA-3′ and Rev 5′-CAACTACGGATATATAAGRRCCRRAACTG-3′) was used as an endogenous control (Adams et al., 2013). This master mix also contained ROX, a passive reference dye, which served as an internal reference for normalization of the SYBR Green I fluorescent signal and allowed for correction of well-to-well variation owing to pipetting inaccuracies and fluorescence fluctuations. The PCR conditions were as follows: initial denaturation at 95 °C for 3 min, followed by 40 cycles at 95 °C for 10 s and 56 °C for 30 s. Data were collected during the annealing step (at 56 °C). For each plate, a no-template control containing all reagents for reverse transcription except for cDNA template was used to check for reagent contamination, and positive controls containing cDNA obtained from plants known to be infected with either CBSV or both CBSV and UCBSV were also used. cDNA synthesized from the RNA of tissue-cultured plants free from both strains was used as a negative control in each plate. Finally, Ct threshold cycle values were obtained and used for the determination of the fold changes in the target genes for both CBSV and UCBSV by using the comparative Ct (2^−ΔΔCt^) method (Livak and Schmittgen, 2001). The fold-change data were subjected to square root transformation to normalize them before performing analysis of variance. Data on disease severity, incidence, and viral load were subjected to analysis of variance performed using Genstat 13th edition, and comparisons of means were made using Fisher's LSD to establish whether there were significant differences among clones.

## 3 Results

### 3.1 CBSD and CMD expression in advanced cassava clones at UYT

**Fig. 1:**
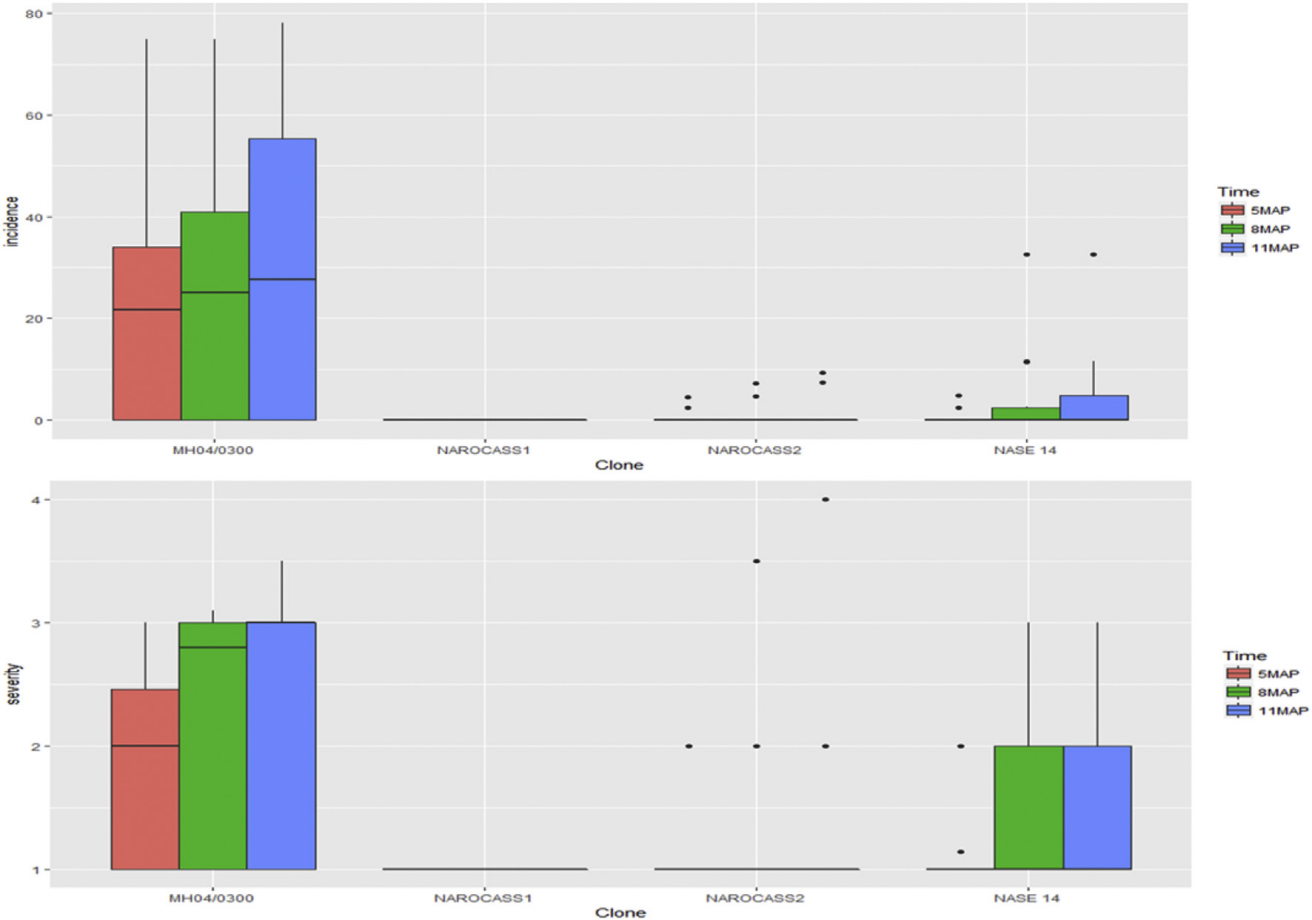
Variability and distribution of CBSD severity and incidence with time among cassava clones of the uniform yield trial.

**Fig. 2:**
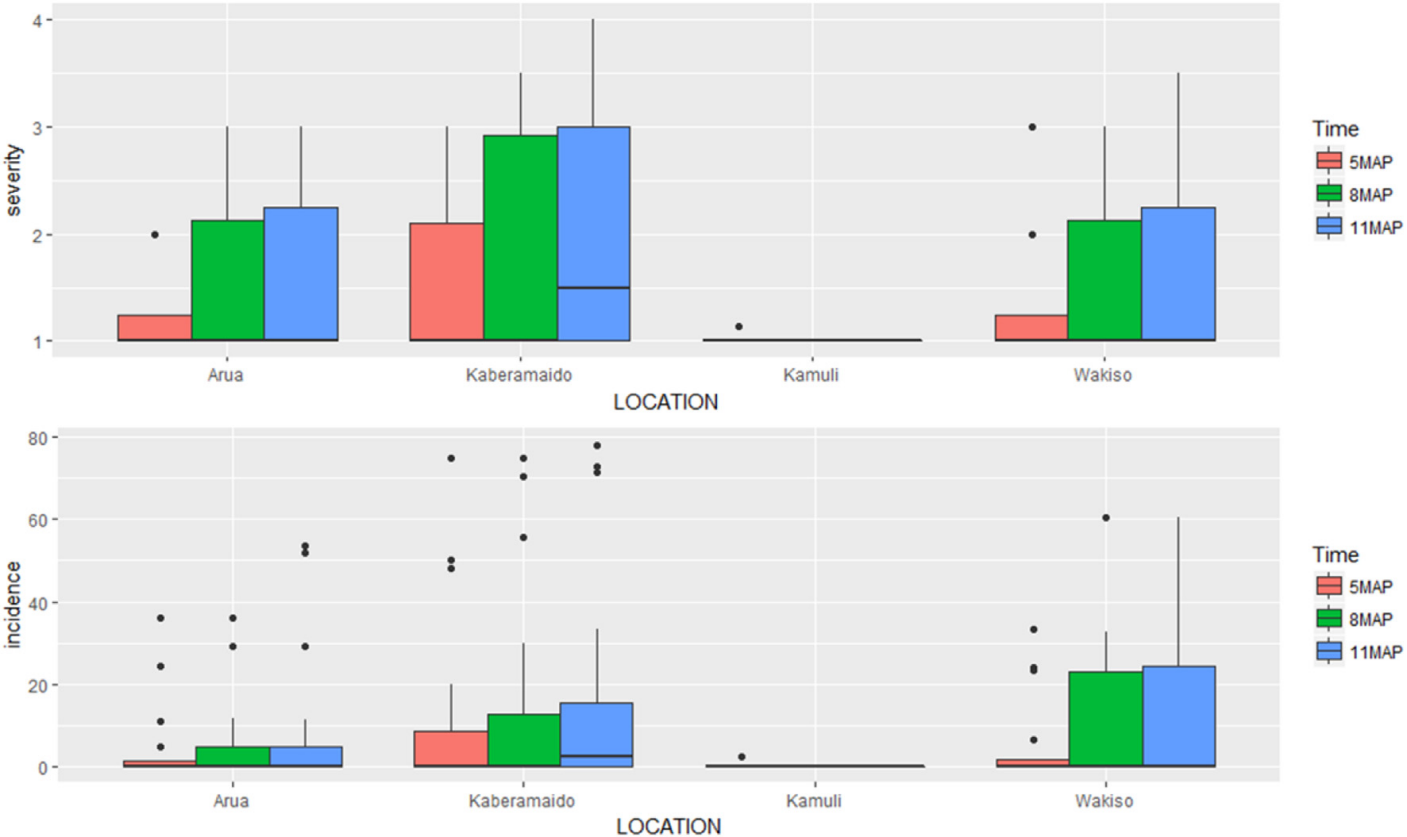
Progress in CBSD incidence (%) and severity of cassava in uniform yield trial locations at 5, 8, and 11 months after planting.

Incidence and severity of CBSD in UYT clones were determined in four locations. CBSD incidence was low in NAROCASS 1 (0%) and NAROCASS 2 (1%), but MH04/0300 had a mean incidence of 33.1% (Table 1). Disease severity was similarly very low in the test clones NAROCASS 1 and NAROCASS 2 compared to that in MH04/0300 (Fig. 1). Notably, CBSD symptoms did not appear on any clone in Kamuli, whereas mild to severe symptoms were recorded in other locations. Indeed, NAROCASS 1 remained symptomless, but NAROCASS 2 had low CBSD incidence that ranged between 0 and 4.6%, with an increasing trend across all locations at 5, 8, and 11 MAP (Fig. 2). By contrast, the clone MH04/0300 had moderate to high CBSD incidence (33.7–63.9%) in all test locations except Kamuli, with the highest incidence (63.9%) recorded at 11 MAP (Fig. 2).

**Table 1:** Mean incidence (%) of cassava brown streak disease on cassava clones for uniform yield trials, 11 months after planting.

Clone	Location				Mean	l.s.d.	F. pr.
	Arua	Kaberamaido	Kamuli	Wakiso			
MH04/0300	33.7	63.9	0	34.8	33.1	28.76	0.004
NAROCASS 1	0	0	0	0	0	0	
NAROCASS 2	0	4.2	0	0	1	3.748	0.078
NASE 14	4.1	2.4	0	11.1	4.4	12.64	0.308
Mean	9.4	17.6	0	11.5	9.6	7.3	<0.001
l.s.d.	19.69	16.44	0	18.52	7.3	14.6	
F. pr.	0.007	<0.001		0.004	<0.001		<0.001

l.s.d. is the least significant difference at *P* = 0.05.

F. pr. is the probability of obtaining the observed variance ratio (*P* value).

Overall, there were significant differences (*P* < 0.05) between test clones for CBSD severity across all locations, except for Kamuli (Table 2). The lowest CBSD mean severity was recorded in Wakiso (1.8) and the highest was in Kaberamaido (2.37). The clone MH04/0300 recorded the highest CBSD severity (3.5), and NAROCASS 1 and NAROCASS 2 had the lowest severity. Among clones that showed CBSD symptoms, NAROCASS 2 had the lowest mean severity (2.5) (Table 2). There were no CMD symptoms observed on any cassava plant in all the UYT locations during the entire study period. This was despite the presence of whitefly vectors and CMD-affected landraces near the trial sites, which would ensure transmission of CMBs.

**Table 2:** Mean severity of cassava brown streak disease on cassava clones for uniform yield trials, 11 months after planting.

Clone	Location				Mean	l.s.d.	c.v. (%)	F. pr.
	Arua	Kaberamaido	Kamuli	Wakiso				
MH04/0300	3.0	3.5	1.0	3.3	2.688	0.6208	14.8	<0.001
NAROCASS 1	1.0	1.0	1.0	1.0	1	0	0	0
NAROCASS 2	1.0	2.5	1.0	1.0	1.375	0.3523	16.3	<0.001
NASE 14	2.3	2.5	1.0	2.0	1.96	0.683	20.8	0.005
Mean	1.833	2.37	1.0	1.812	1.755	0.2088		<0.001
l.s.d.	0.4068	0.894	0	0.4315	0.2088	0.4177		
c.v. (%)	14.1	22.5	0	15.1			16.7	
F. pr.	0.014	0.002	0	<0.001	<0.001			<0.001

l.s.d. is the least significant difference at *P* = 0.05.

c.v. is the coefficient of variation.

F. pr. is the probability of obtaining the observed variance ratio (*P* value).

### 3.2 Incidence of CBSVs in samples collected from clones in UYTs

In the assay of 88 RNA extracts from leaves of CBSD symptomatic plants, 68 (77.3%) showed a positive result for CBSVs (Table 3). Of the 68 CBSV-positive samples, 61 (89.7%) had single CBSV infections and seven (10.3%) were infected with both CBSV and UCBSV (Table 3). Interestingly, when 360 samples from non-symptomatic plants were analyzed, 30 plants (8.3%) showed a positive result for CBSVs. Among the 30 CBSV-positive samples from the non-symptomatic group, 23 (76.7%) had CBSV single infections and seven (23.3%) were infected with both CBSV and UCBSV. CBSV infection was highest (100%) in Kaberamaido followed by Arua (60%). Mixed infections of CBSV and UCBSV were recorded only in 14 samples in two locations: Arua and Wakiso. The single infections with CBSV were highest in the clone MH04/0300 (65.5%), followed by NASE 14 (27.4%) and NAROCASS 2 (7.1%; Table 3).

**Table 3:** Prevalence of CBSVs in samples collected from cassava clones at uniform yield trial locations.

Symptomatic						Nonsymptomatic				
Location	Clone	a	CBSV	UCBSV	CBSV + UCBSV	a	CBSV	UCBSV	CBSV + UCBSV	Total positive (%)
Arua	MH04/0300	19	10	0	6	9	3	0	5	24 (96)
	NAROCASS 1	0	0	0	0	28	0	0	0	0 (0)
	NAROCASS 2	2	1	0	0	26	1	0	0	2 (8)
	NASE 14	4	3	0	1	24	0	0	0	4 (16)
Kaberamaido	MH04/0300	21	16	0	0	7	7	0	0	23 (92)
	NAROCASS 1	0	0	0	0	28	0	0	0	0 (0)
	NAROCASS 2	4	2	0	0	24	2	0	0	4 (16)
	NASE 14	12	8	0	0	16	2	0	0	10 (40)
Kamuli	MH04/0300	0	0	0	0	28	0	0	0	0 (0)
	NAROCASS 2	0	0	0	0	28	0	0	0	0 (0)
	NAROCASS 1	0	0	0	0	28	0	0	0	0 (0)
	NASE 14	0	0	0	0	28	0	0	0	0 (0)
Wakiso	MH04/0300	17	14	0	0	11	5	0	2	21 (84)
	NAROCASS 1	0	0	0	0	28	0	0	0	0 (0)
	NAROCASS 2	0	0	0	0	28	0	0	0	0 (0)
	NASE 14	9	7	0	0	19	3	0	0	10 (40)
Total		88	61	0	7	360	23	0	7	98 (20.08)

a – Number of samples.

### 3.3 CBSV and UCBSV titers in leaf samples from UYT clones

**Fig. 3:**
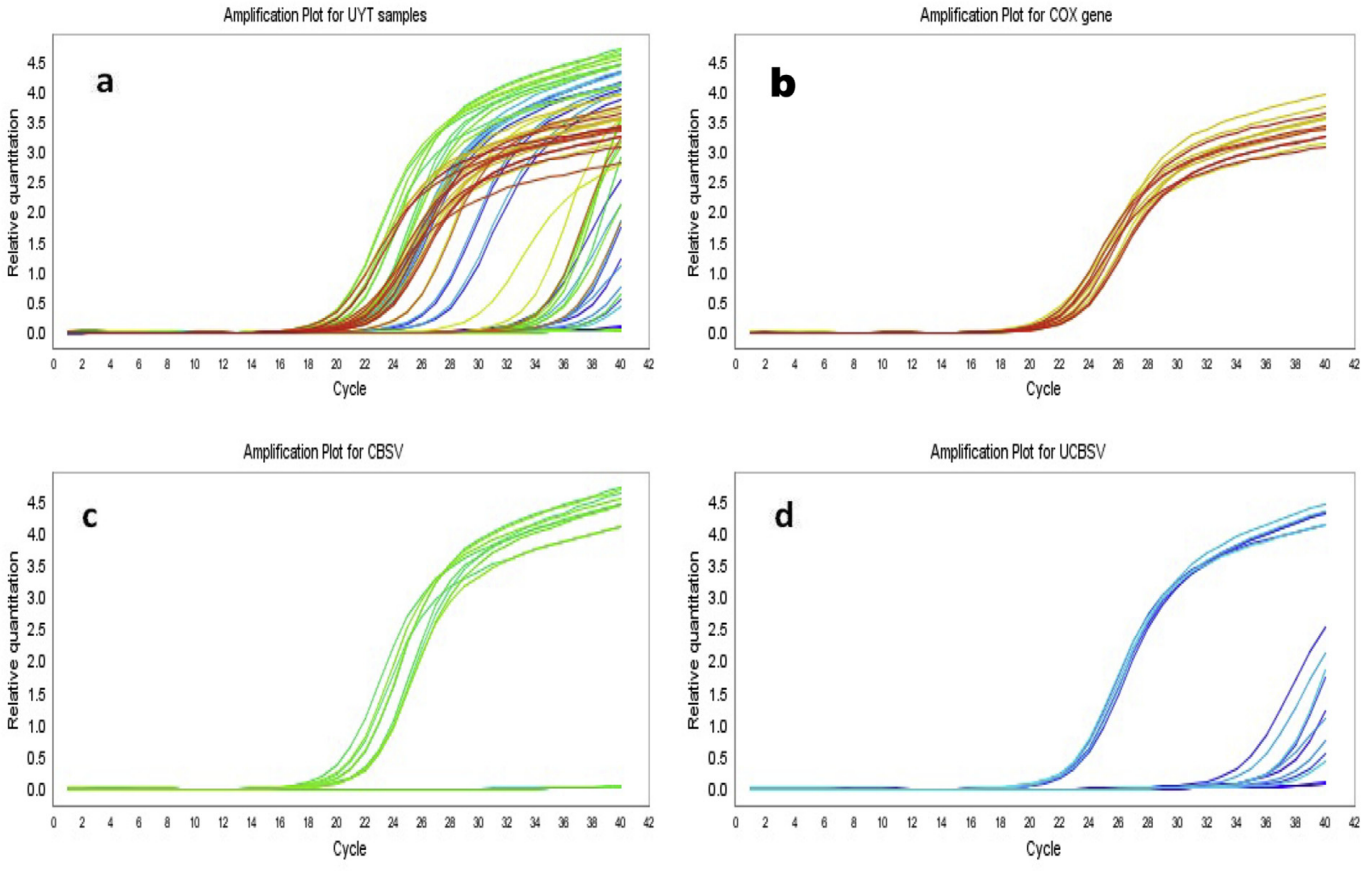
Amplification plots for (a) CBSV, UCBSV, and *COX*; (b) *COX*, the housekeeping gene; (c) CBSV; and (d) UCBSV.

NAROCASS 1 and NAROCASS 2 had the lowest viral load compared with other clones evaluated in this study. CBSV was not detected in NAROCASS 1 across all locations, whereas UCBSV was present in low concentrations in NAROCASS 1 in Arua (5 MAP) and Kaberamaido (5 and 11 MAP) (Table 4, Table 5). In general, virus concentration increased with the age of plants (5–11 MAP) for both CBSV and UCBSV. However, interestingly, for MH04/0300 (Arua), NAROCASS 2 (Kaberamaido, Kamuli and Wakiso), and NAROCASS 1 (Arua), the UCBSV titers decreased with time. For example, in Arua, UCBSV titer decreased from 3.56- to 2.15-fold in the clone MH04/0300 (Table 5). By contrast, CBSV concentration increased sharply for the clone MH04/0300 in Arua, Kaberamaido, and Wakiso (Table 4). In Kaberamaido, where CBSD incidences were high, MH04/0300 accumulated the most CBSV RNA (6.71- and 20.56-fold) compared with the tolerant NASE 14 (0.58- and 7.32-fold) at 5 and 11 MAP, respectively. The amplification plot of cycle number versus relative quantitation showed that many CBSV samples had an early threshold cycle (Fig. 3).

**Table 4:** CBSV titers (fold changes) in cassava genotypes for uniform yield trials at 5 and 11 months after planting (MAP).

Clone	Location							
	Arua		Kaberamaido		Kamuli		Wakiso	
	5 MAP	11 MAP	5 MAP	11 MAP	5 MAP	11 MAP	5 MAP	11 MAP
MH04/0300	0.377	12.19	6.706	20.56	0.109	0.91	0.579	9.04
NAROCASS 1	0.04	0.02	0.064	0.02	0.008	0.01	0.018	0.01
NAROCASS 2	0.032	0.06	0.089	1.4	0.011	0.01	0.024	0.76
NASE 14	0.132	5.53	0.578	7.32	0.03	0.04	0.114	1.56
Mean	0.145	4.45	1.859	7.32	0.04	0.24	0.184	2.84
l.s.d.	0.3218	2.971	0.89	3.706	0.0997	1.713	0.849	4.321
F pr	0.113	<0.001	<0.001	<0.001	0.133	0.467	0.337	0.013

l.s.d. is the least significant difference at *P* = 0.05.

F. pr. is the probability of obtaining the observed variance ratio (*P* value).

**Table 5:** UCBSV titers (fold changes) in cassava genotypes for uniform yield trials at 5 and 11 months after planting (MAP).

Clone	Location							
	Arua		Kaberamaido		Kamuli		Wakiso	
	5 MAP	11 MAP	5 MAP	11 MAP	5 MAP	11 MAP	5 MAP	11 MAP
MH04/0300	3.56	2.15	14.1	20.1	0.16	0.43	0.03	0.055
NAROCASS 1	0.67	0.06	0.1	0.2	0.03	0.022	0.04	0.006
NAROCASS 2	0.03	0.01	0.1	0	0.36	0.003	0.87	0.156
NASE 14	0.28	12.96	0	4.6	0.14	0.238	0.06	0.057
Mean	1.13	3.79	3.6	6.2	0.17	0.173	0.25	0.068
l.s.d.	5.726	2.554	27.69	12.27	0.788	0.4913	1.551	0.2604
F pr	0.399	<0.001	0.481	0.029	0.712	0.19	0.446	0.518

l.s.d. is the least significant difference at *P* = 0.05.

F. pr. is the probability of obtaining the observed variance ratio (*P* value).

### 3.4 CBSD root symptom incidence, severity, and yield performance of cassava clones

The test materials recorded low CBSD root incidence in all locations except in the clone MH04/0300, which registered the highest root incidence of 68% in Wakiso (Table 6). Mean CBSD root severity was also significantly lower (*P* < 0.05) in the clones NAROCASS 1 (2.0) and NAROCASS 2 (2.0) than in the clone MH04/0300 (3.8) in Wakiso. The maximum CBSD root severity ranged from 4 to 5 for NASE 14 and MH04/0300 in Kaberamaido, Arua, and Wakiso, whereas in Kamuli, the maximum CBSD root severity was for NASE 14 with a score of 2.8.

**Table 6:** Cassava brown streak disease root incidence, mean severity, maximum severity, harvest index, and yield performance of clones at uniform yield trial locations.

	Clone	CBSDRi	Mean_CBSDRs	max_CBSDRs	Harvest_Index	Yield_T_ha
Kaberamaido	MH04/0300	28.3	1.8	4.0	0.5	7.4
	NAROCASS 1	2.3	1.0	2.0	0.7	22.8
	NAROCASS 2	3.7	1.1	3.0	0.6	13.9
	NASE 14	12.5	1.4	4.5	0.5	22.2
	mean	11.7	1.3	3.4	0.6	16.6
	l.s.d.	26.9	0.8	2.0	0.2	6.6
	cv%	128.2	35	41	20.3	28.2
	F pr.	0.106	0.11	0.103	0.282	0.001
Kamuli	MH04/0300	0.6	1.0	1.3	0.5	14.8
	NAROCASS 1	1.9	1.0	2.5	0.5	28.9
	NAROCASS 2	1.9	1.0	1.8	0.5	16.7
	NASE 14	3.8	1.1	2.8	0.5	30.8
	mean	2.1	1.0	2.1	0.5	22.8
	l.s.d.	2.3	0.1	1.6	0.1	11.9
	cv%	70.2	3.2	50	13.2	32.6
	F pr.	0.073	0.267	0.22	0.214	0.027
Arua	MH04/0300	9.8	1.1	4.0	0.6	31.0
	NAROCASS 1	0.2	1.0	1.3	0.6	53.3
	NAROCASS 2	4.6	1.1	2.5	0.6	20.9
	NASE 14	7.5	1.2	5.0	0.6	34.2
	mean	5.2	1.1	3.1	0.6	34.9
	l.s.d.	7.1	0.1	1.2	0.1	22.9
	cv%	84.2	6.2	23.1	6	42.7
	F pr.	0.065	0.018	<0.001	0.039	0.057
Wakiso	MH04/0300	68.0	3.8	5.0	0.6	39.4
	NAROCASS 1	3.9	2.0	4.0	0.5	55.6
	NAROCASS 2	4.5	2.1	3.0	0.4	23.3
	NASE 14	41.9	3.1	5.0	0.4	42.3
	mean	29.6	2.8	4.3	0.5	40.2
	l.s.d.	60.4	1.7	2.8	0.2	20.3
	cv%	73.5	22.7	23.5	13.1	18.2
	F pr.	0.103	0.113	0.281	0.115	0.05

CBSDRi = cassava brown streak disease root incidence, Mean_CBSDRs = mean cassava brown streak disease root severity, max_CBSDRs = maximum cassava brown streak disease root severity, Yield_T_ha = yield in tonnes per hectare, l.s.d. = least significant difference at *P* = 0.05, c.v. = coefficient of variation, F. pr. = probability of obtaining the observed variance ratio (*P* value).

The HI ranged between 0.4 and 0.7 for all the clones across the locations and was the highest for NAROCASS 1 at Kaberamaido (0.7) and the lowest for NAROCASS 2 (0.4) and NASE 14 (0.4) in Wakiso. However, little variation was observed in HI for clones in Arua and Kamuli (Table 6).

NAROCASS 1 and NASE 14 had the highest yield in all locations. Yields of up to 55.6 and 53.3 t/ha were obtained for clone NAROCASS 1 in Wakiso and Arua, respectively. The lowest yields (14 t/ha) were obtained for the clone MH04/0300 in Kaberamaido.

### 3.5 Expression of CMD and CBSD on cassava genotypes in CBT

**Fig. 4:**
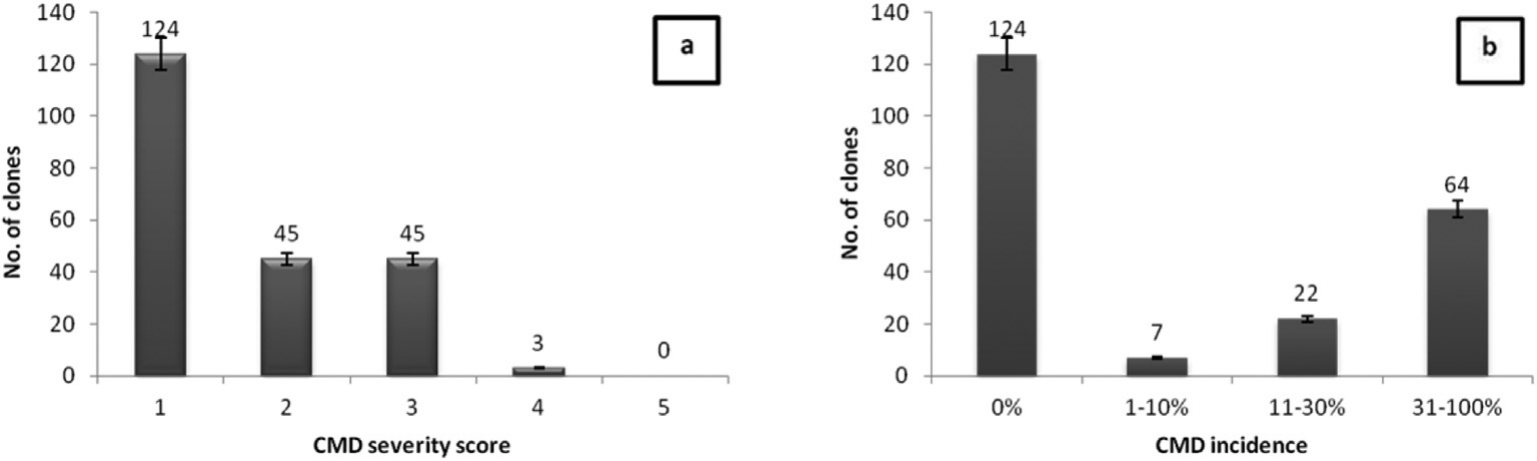
CMD severity (a) and incidence (b) at 12 months after planting on 217 genotypes assessed in the crossing block trial.

CMD was observed in 93 (42.9%) of the 217 clones evaluated in the CBT. However, CMD was not recorded in 124 (57.1%) of the clones throughout the evaluation period. There were significant differences (*P* < 0.05) between the test clones. Seven clones had CMD incidence in the range of 1–10%, whereas 22 and 64 clones had incidences ranging within 11–30% and 31–100%, respectively (Fig. 4b). The affected cassava plants mainly exhibited mild to moderate CMD symptom severity scores of 2 and 3 (Fig. 4a).

**Fig. 5:**
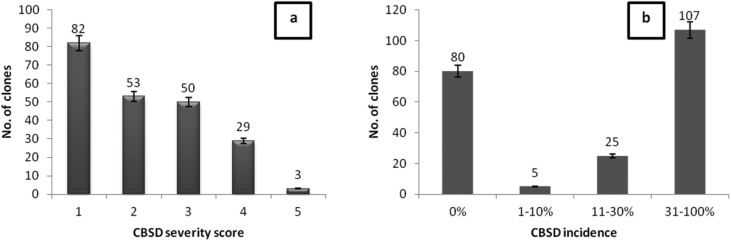
CBSD severity (a) and incidence (b) at 12 months after planting on 217 genotypes assessed in the crossing block trial.

In the CBT, 83 (38.2%) clones did not show any CBSD symptoms (0% incidence). Most (102) of the clones (47%) had CBSD incidence in the range of 31–100%, whereas incidences in the remaining 26 and 6 clones were in the ranges of 10–21% and 1–10%, respectively (Fig. 5b). Of the 134 CBSD-affected clones, 51 showed mild CBSD symptoms (severity score 2) at most. A total of 50 clones had a score of 3, and 33 clones were severely affected (scores 4 and above) (Fig. 5a).

### 3.6 Detection of CBSVs and CMBs in cassava clones in the CBT

**Fig. 6:**
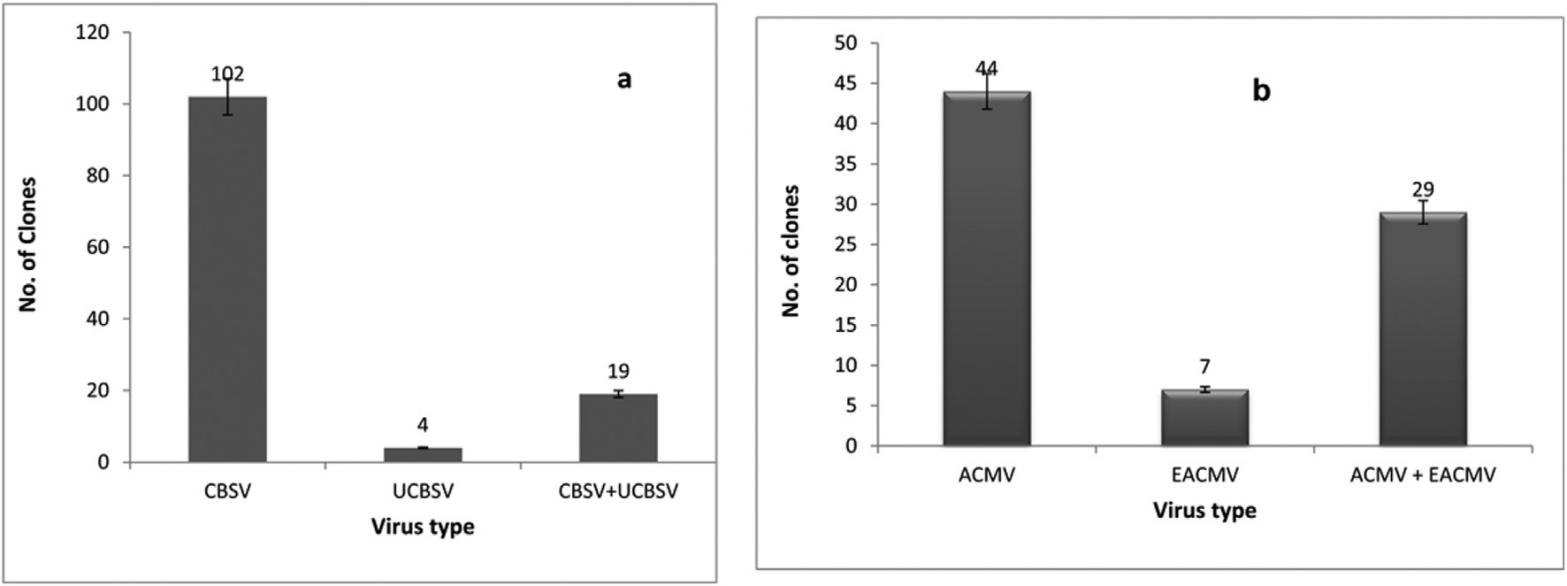
Detection of (a) CBSVs and (b) CMBs in the cassava clones evaluated in the crossing block trial.

Single CBSV infections were predominant in the CBT, for which 102 (81.6%) of 125 clones showed a positive result for CBSVs. Only four (3.2%) clones had single UCBSV infections, whereas 19 (15.2%) had mixed infections of both CBSV and UCBSV (Fig. 6a). There were 80 samples that tested positive for CMBs; 44 (55%) had ACMV alone, seven (8.7%) had EACMV-UG single infection, and 29 (36.3%) were dually infected with ACMV and EACMV-UG (Fig. 6b).

## 4 Discussion

We assessed the reaction of 220 elite cassava germplasm for resistance to CBSD and CMD in four UYT locations (Arua, Kamuli, Kaberamaido, and Wakiso) and in the CBT located at Wakiso, Uganda, in 2014, where the two viral diseases have caused significant constraints to food and income security. The cassava breeding program in Uganda has successfully selected varieties for CMD resistance during the last two decades; thus, the clones evaluated at UYT can be considered to possess such pedigree. Absence of CMD in all the test clones present at UYT suggests that these clones have an excellent background of resistance to the disease. Effectively, the clones NAROCASS 1, NAROCASS 2, NASE 14, and MH04/0300 present an invaluable genetic resource that could be used to generate breeding populations for enhanced resistance to CMD. However, CMD symptoms were observed on 61.8% of the clones evaluated in the CBT. The overall CMD severity was only mild to moderate with the majority of symptomatic plants scoring between 2 and 3, which further illustrates the remarkable progress in genetic improvement of cassava for disease resistance (Kawuki et al., 2016).

In this study, CMBs were detected by PCR and the presence of CBSVs and associated virus concentrations determined by RT-PCR and real-time qPCR, respectively. Molecular detection of CMBs in the CBT indicated single and dual infections with ACMV and EACMV-UG. These results demonstrated that ACMV was the most prevalent (55%) virus species followed by the mixed infection of ACMV + EACMV-UG (26%) and then EACMV (6%). These results are interesting as they contrast the situation of the CMD epidemic in the 1990s when EACMV-UG was the most prevalent species causing CMD (Duffy and Holmes, 2009). However, in coping with the CMD epidemic, farmers may have selected less-symptomatic plants, which effectively reduced the incidence of EACMV-UG. In addition, the severe symptoms of CMD caused by EACMV-UG probably made culling of genotypes carrying this species easier, hence likely reducing the overall EACMV-UG prevalence.

Variations in CBSD severity were consistent with those reported in other studies (Hillocks and Jennings, 2003). Notably, however, NAROCASS 1 did not show any CBSD symptoms in UYT and NAROCASS 2 had only mild symptoms. The absence of CBSD symptoms on NAROCASS 1 and the low incidence and severity on NAROCASS 2 demonstrate high levels of resistance and/or tolerance to the disease. Moreover, NAROCASS 1 and NAROCASS 2 also had the lowest virus concentrations across all sites, thus corroborating the earlier deduction that these clones were superior sources of resistance to CBSD. In fact, Kaweesi et al. (2014) and Maruthi et al. (2014) indicated that some cassava cultivars do hinder normal replication of CBSVs in tissues, which could partly explain the low viral load and mild symptom expression. Therefore, breeding programs could target using such cultivars, with the ability to inhibit or delay the rate of viral replication in their plant systems, for developing resistant cassava varieties in the future.

Additionally, both RT-PCR and qPCR results revealed the single infections of CBSV to be the most prevalent. In the CBT, CBSV single infections occurred in 84.3% of the infected clones, thus suggesting that CBSV was the most prevalent virus species. Mixed infections were detected only in Arua (40%) and Wakiso (6.5%). In general, 85.7% of the positive samples had CBSV, no sample had single infection by UCBSV, and only 14.3% had mixed infections. Similar trends have been reported from initial studies aimed at screening large sets of cassava genetic resources for resistance to CBSD (Abaca et al., 2012). It is logical to suggest that certain cassava genotypes have inherent mechanisms to restrict the activities of UCBSV in cells; such clones would be of interest in developing CBSD-resistant varieties.

Variations in cassava yields were recorded in the UYT plant materials, with NAROCASS 1 obtaining higher yields (55.6 t/ha) than the other clones. Kaberamaido had notably the lowest yields recorded compared to other locations. The variation exhibited by UYT clones in response to disease parameters agrees with other findings by Hillocks and Jennings (2003) that the susceptibility or resistance of varieties influences their response to disease infection. By contrast, the yield was relatively lower in Kamuli, where clones had a low viral load as well as foliar and root CBSD incidence and severity, than those in other locations. This was probably due to stacked resistant genes in the plants that masked more the expression of genes responsible for yield in this location compared to other UYT environments.

Equally important to note was the severity of CBSD in NASE 14, a variety officially released in 2011 for its tolerance to this disease. NASE 14 is well known for its ability to restrict build-up of viral load (Kaweesi et al., 2014). However, the current study revealed relatively high foliar and root severity, incidence, and viral load in this clone. It is unknown why such behavior was manifested, and hence, there is a need to study the mechanism involved in resistance breakdown of such varieties. This phenomenon is worrisome in that varieties released to address specific viral diseases may not be sufficiently resistant to provide an effective long-term control (Kawuki et al., 2016). Inevitably, greater efforts are needed to consolidate breeding activities toward the development of varieties with strong and durable resistance to CBSD, which may call for renewed regional collaboration for the use of elite germplasm. Additionally, more efforts are needed to build capacity for the development and implementation of seed systems for cassava to ensure sustainable supply of disease-free planting materials of improved varieties to farmers.

## 5 Conclusion

CMD and CBSD remain a major threat to cassava production in eastern Africa, although the impact of the former has been significantly reduced during the last two decades through dissemination of resistant clones. In addition to NAROCASS 1 and NAROCASS 2, several other clones evaluated according to disease incidence, symptom severity, and viral load in this study showed high levels of resistance to CBSD. These clones should be of priority when implementing breeding programs to develop and deploy resistant varieties. Differential clone response to CBSD by location was observed, and this should be considered when prescribing approaches for CBSD management. This requires that genotype × environment × time interaction studies be conducted in further field evaluations for a long period. Additionally, studies based on symptoms need to be augmented by molecular diagnostics and plant–virus interaction analysis for better CBSD assessment. We recommend continuous monitoring of CBSD symptom variability and how these may relate to potential emergence of virus strains or species, a strategy necessary to avert rapid breakdown of resistance in varieties or degeneration of planting materials.
